# Transparency and openness in science

**DOI:** 10.1098/rsos.160979

**Published:** 2017-01-11

**Authors:** Jeremy Sanders, Jon Blundy, Anne Donaldson, Steve Brown, Rob Ivison, Miles Padgett, Kevin Padian, Katrin Rittinger, Kerry Rowe, Anthony Stace, Essi Viding, Chris Chambers, Mark Chaplain

**Affiliations:** 1 Department of Chemistry, University of Cambridge, Cambridge, CB2 1EW, UK; 2 School of Earth Sciences, University of Bristol, Bristol, UK; 3 Institute of Medical Sciences, University of Aberdeen, Aberdeen AB25 2ZD, UK; 4 Mammalian Genetics Unit, MRC Harwell Institute, Harwell OX11 0RD, UK; 5 Institute for Astronomy, University of Edinburgh, Edinburgh EH9 3HJ, UK; 6 European Southern Observatory, Munich, Germany; 7 Department of Physics and Astronomy, University of Glasgow, Glasgow, UK; 8 Museum of Paleontology, University of California, Berkeley, CA 94720-4780, USA; 9 The Francis Crick Institute, London NW7 1AA, UK; 10 Department of Civil Engineering, Queen's University, Kingston, Ontario, CanadaK7 L 3N6; 11 School of Chemistry, University of Nottingham, Nottingham, UK; 12 UCL Division of Psychology and Language Sciences, University College London, London, UK; 13 School of Psychology, Cardiff University, Cardiff CF10 3AT, UK; 14 School of Mathematics and Statistics, University of St Andrews, St Andrews KY16 9SS, UK

*Royal Society Open Science* has been in the vanguard of efforts to improve both transparency and reproducibility in science, since its launch in 2014.

Among the wider scientific community, there is a widespread dissatisfaction with the current level of transparency and reproducibility in published research and, as part of our response to this, we signed up to the *Transparency and Openness Promotion* (*TOP*) *Guidelines* (https://cos.io/top/).

The objective of the *TOP guidelines* is to encourage transparency, openness and reproducibility in science. By developing shared standards for openness across journals, it is hoped to change the current incentive structures to drive researchers' behaviour towards more openness.

The *TOP guidelines* help achieve this through journals' procedures and policies for publication. As a TOP signatory journal, we have conducted a review of the guidelines to ensure their effective adoption. This review was conducted with input from senior editors who agreed on appropriate levels of adoption for each standard.

The senior editors at *Royal Society Open Science* believe that many of the guidelines provide a sensible standard for scientists to follow when planning their research and preparing their manuscripts for publication, and that is why we have chosen to publish the standards we are adopting.

Many of our authors are already applying these practices. We would like to encourage those who do not follow them to do so. We will continue to publish well-conducted research of broad appeal. However, we are keen to promote best practice, and the guidelines described in this editorial are one way to help us do this.

The guidelines are based on eight standards. Three levels of adoption are recognized from 1 (most lenient) to 3 (most stringent). *Royal Society Open Science* will adhere to the following TOP guideline levels:

*Citation standards.* Level 2: article provides appropriate citation for data, code and materials used consistent with journal's author guidelines.
*Data transparency.* Level 2: data must be posted to a trusted repository. Exceptions must be identified at article submission.
*Analytic methods (code) transparency.* Level 2: novel code must be posted to a trusted repository. Exceptions must be identified at article submission.
*Digital research materials transparency.* Level 2: article states whether digital materials are available and, if so, where to access them.
*Design and analysis transparency.* Level 1: journal articulates design transparency standards.
*Pre-registration of studies.* Level 1: journal encourages pre-registration of studies, if relevant, and provides link in article to pre-registration if it exists.
*Pre-registration of analysis plans.* Level 1: journal encourages pre-analysis plans, if relevant, and provides link in article to registered analysis plan if it exists.
*Replication.* Level 3: journal uses Registered Reports as a submission option for replication studies with peer review prior to observing the study outcomes.

The standards we have set at Level 1 recognizes that preregistration is not a normal practice in many scientific disciplines—so we only encourage pre-registration. The standards we have set at Level 2 reflect the current Royal Society policies on citation standards and transparency. We are leading the way as a journal which provides the option of Registered Reports across all of science for replication.

